# Diethyl 4,4′-(diazenediyl)dibenzoate

**DOI:** 10.1107/S1600536811036877

**Published:** 2011-09-17

**Authors:** Yongsheng Niu, Jianping Huang, Chunpu Zhao, Pan Gao, Youzhu Yu

**Affiliations:** a Department of Chemistry and Environmental Engineering, Anyang Institute of Technology, Henan 455000, People’s Republic of China

## Abstract

The full mol­ecule of the title compound, C_18_H_18_N_2_O_4_, is generated by the application of an inversion centre. There are strong π–π inter­actions between adjacent mol­ecules with a centroid–centroid distance of 3.298 (2)Å.

## Related literature

For the properties and structures of related compounds, see: Altomare *et al.* (2005 ▶;) Kubo *et al.* (2005 ▶); Harada *et al.* (1997 ▶).
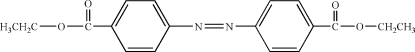


## Experimental

### 

#### Crystal data

C_18_H_18_N_2_O_4_
*M* *_r_* = 326.34
Monoclinic, 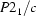

*a* = 14.844 (3) Å
*b* = 4.5731 (9) Å
*c* = 11.814 (2) Å
β = 95.88 (3)°
*V* = 797.7 (3) Å^3^
*Z* = 2
Mo *K*α radiation
μ = 0.10 mm^−1^
*T* = 293 K
0.40 × 0.20 × 0.20 mm

#### Data collection

Bruker APEXII CCD diffractometer
13606 measured reflections
1976 independent reflections
1639 reflections with *I* > 2σ(*I*)
*R*_int_ = 0.022

#### Refinement

*R*[*F* ^2^ > 2σ(*F* ^2^)] = 0.050
*wR*(*F* ^2^) = 0.149
*S* = 1.07
1976 reflections
109 parameters
H-atom parameters constrained
Δρ_max_ = 0.31 e Å^−3^
Δρ_min_ = −0.25 e Å^−3^

### 

Data collection: *APEX2* (Bruker, 2005 ▶); cell refinement: *SAINT-Plus* (Bruker, 2001 ▶); data reduction: *SAINT-Plus*; program(s) used to solve structure: *SHELXS97* (Sheldrick, 2008 ▶); program(s) used to refine structure: *SHELXL97* (Sheldrick, 2008 ▶); molecular graphics: *SHELXTL* (Sheldrick, 2008 ▶); software used to prepare material for publication: *SHELXTL*.

## Supplementary Material

Crystal structure: contains datablock(s) I, global. DOI: 10.1107/S1600536811036877/aa2020sup1.cif

Structure factors: contains datablock(s) I. DOI: 10.1107/S1600536811036877/aa2020Isup2.hkl

Additional supplementary materials:  crystallographic information; 3D view; checkCIF report

## Figures and Tables

**Fig. 1.**
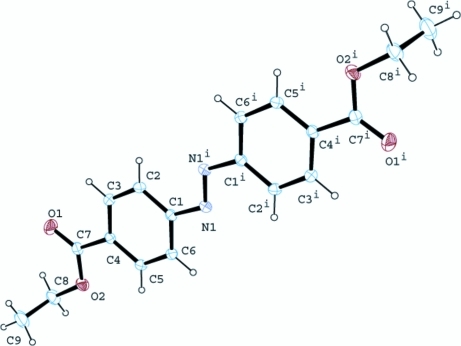
The molecular structure of the title compound, with displacement ellipsoids drawn at the 30% probability level. Symmetry code: (i) 2 - x, - y, 2 - z

**Fig. 2.**
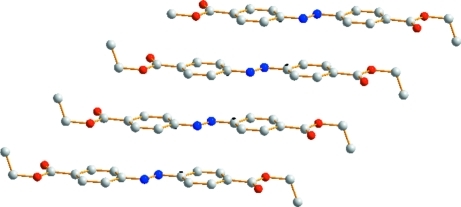
π-π interaction of the molecules.

## supplementary crystallographic information

### Comment

Synthesis, elucidation of crystal structures, and investigation of physical properties of new liquid crystals are important for studying the relationship between molecular stuctures and mesophases. (Kubo *et al.*, 2005). As a contribution to these fields, We report here the synthesis and structure of the title compound.

The title compound (Fig. 1), C_18_H_18_N_2_O_4_, shows crystallographic inversion symmetry. The intersection angle between two benzene rings is consistent with that of azobenzene (0 °). (Harada *et al.*, 1997) No classic hydrogen bonds are observed in the crystal. There are strong π-π interactions between planar adjacent molecules with the interplanar distance 3.298 Å.

### Experimental

1.0 g of azobenzene-4,4'-dicarbonylchloride and 20 ml of ethanol were stirred at 353 K for 4 h. After cooling to room temperature a red deposit was obtained. It was then recrystallized from CH_2_Cl_2_ to give red crystals suitable for X-ray diffraction analysis.

### Refinement

All H atoms were placed in geometrically idealized positions (C—H = 0.93, 0.96 and 0.97 Å) and treated as riding on their parent atoms with *U*_iso_(H) = 1.2*U*_eq_(C) and 1.5*U*_eq_(C).

### Crystal data

**Table:**

C_18_H_18_N_2_O_4_	*F*(000) = 344
*M**_r_* = 326.34	*D*_x_ = 1.359 Mg m^−^^3^
Monoclinic, *P*2_1_/*c*	Mo *K*α radiation, λ = 0.71073 Å
Hall symbol: -P 2ybc	Cell parameters from 5335 reflections
*a* = 14.844 (3) Å	θ = 3.5–28.4°
*b* = 4.5731 (9) Å	µ = 0.10 mm^−^^1^
*c* = 11.814 (2) Å	*T* = 293 K
β = 95.88 (3)°	Block, red
*V* = 797.7 (3) Å^3^	0.40 × 0.20 × 0.20 mm
*Z* = 2	

### Data collection

**Table:**

Bruker APEXII CCD diffractometer	1639 reflections with *I* > 2σ(*I*)
Radiation source: fine-focus sealed tube	*R*_int_ = 0.022
graphite	θ_max_ = 28.4°, θ_min_ = 3.5°
φ and ω scans	*h* = −19→19
13606 measured reflections	*k* = −6→6
1976 independent reflections	*l* = −15→15

### Refinement

**Table:**

Refinement on *F*^2^	Primary atom site location: structure-invariant direct methods
Least-squares matrix: full	Secondary atom site location: difference Fourier map
*R*[*F*^2^ > 2σ(*F*^2^)] = 0.050	Hydrogen site location: inferred from neighbouring sites
*wR*(*F*^2^) = 0.149	H-atom parameters constrained
*S* = 1.07	*w* = 1/[σ^2^(*F*_o_^2^) + (0.0736*P*)^2^ + 0.2526*P*] where *P* = (*F*_o_^2^ + 2*F*_c_^2^)/3
1976 reflections	(Δ/σ)_max_ < 0.001
109 parameters	Δρ_max_ = 0.31 e Å^−^^3^
0 restraints	Δρ_min_ = −0.25 e Å^−^^3^

### Fractional atomic coordinates and isotropic or equivalent isotropic displacement parameters (Å^2^)

**Table:**

	*x*	*y*	*z*	*U*_iso_*/*U*_eq_	
C1	0.92686 (8)	0.2685 (3)	0.93120 (11)	0.0327 (3)	
C2	0.86559 (9)	0.3422 (3)	1.00838 (12)	0.0390 (3)	
H2A	0.8699	0.2558	1.0800	0.047*	
C3	0.92142 (9)	0.4028 (3)	0.82545 (11)	0.0353 (3)	
H3A	0.9633	0.3565	0.7748	0.042*	
C4	0.85380 (9)	0.6061 (3)	0.79487 (11)	0.0360 (3)	
H4A	0.8502	0.6954	0.7238	0.043*	
C5	0.79835 (9)	0.5450 (3)	0.97752 (12)	0.0398 (3)	
H5A	0.7573	0.5946	1.0288	0.048*	
C6	0.79149 (8)	0.6754 (3)	0.87074 (11)	0.0340 (3)	
C7	0.71536 (9)	0.8857 (3)	0.84138 (13)	0.0407 (3)	
C8	0.64597 (12)	1.2071 (4)	0.69866 (17)	0.0604 (5)	
H8A	0.6696	1.3527	0.6498	0.072*	
H8B	0.6251	1.3072	0.7635	0.072*	
C9	0.56912 (14)	1.0525 (6)	0.6352 (2)	0.0848 (8)	
H9A	0.5226	1.1908	0.6103	0.127*	
H9B	0.5453	0.9099	0.6838	0.127*	
H9C	0.5896	0.9565	0.5702	0.127*	
N1	0.99820 (7)	0.0589 (2)	0.95208 (9)	0.0354 (3)	
O1	0.65887 (8)	0.9411 (3)	0.90410 (11)	0.0645 (4)	
O2	0.71756 (7)	1.0021 (3)	0.73787 (10)	0.0502 (3)	

### Atomic displacement parameters (Å^2^)

**Table:**

	*U*^11^	*U*^22^	*U*^33^	*U*^12^	*U*^13^	*U*^23^
C1	0.0323 (6)	0.0305 (6)	0.0344 (7)	−0.0026 (5)	−0.0018 (5)	−0.0015 (5)
C2	0.0417 (7)	0.0438 (8)	0.0315 (7)	−0.0004 (6)	0.0032 (5)	0.0052 (5)
C3	0.0359 (6)	0.0373 (7)	0.0329 (7)	0.0013 (5)	0.0039 (5)	−0.0006 (5)
C4	0.0392 (6)	0.0380 (7)	0.0303 (6)	0.0012 (5)	0.0008 (5)	0.0017 (5)
C5	0.0363 (6)	0.0469 (8)	0.0368 (7)	0.0007 (6)	0.0071 (5)	−0.0010 (6)
C6	0.0311 (6)	0.0337 (6)	0.0361 (7)	−0.0019 (5)	−0.0016 (5)	−0.0033 (5)
C7	0.0344 (6)	0.0404 (7)	0.0462 (8)	0.0016 (5)	−0.0005 (5)	−0.0048 (6)
C8	0.0546 (9)	0.0584 (10)	0.0651 (11)	0.0187 (8)	−0.0086 (8)	0.0054 (9)
C9	0.0547 (10)	0.1064 (19)	0.0875 (16)	0.0183 (11)	−0.0209 (10)	−0.0167 (14)
N1	0.0370 (6)	0.0347 (6)	0.0334 (6)	−0.0007 (4)	−0.0009 (4)	0.0008 (4)
O1	0.0515 (7)	0.0768 (9)	0.0673 (8)	0.0215 (6)	0.0171 (6)	0.0054 (7)
O2	0.0468 (6)	0.0546 (7)	0.0475 (6)	0.0157 (5)	−0.0033 (5)	0.0054 (5)

### Geometric parameters (Å, °)

**Table:**

C1—C3	1.3870 (18)	C6—C7	1.4972 (18)
C1—C2	1.3934 (19)	C7—O1	1.2015 (18)
C1—N1	1.4311 (17)	C7—O2	1.3371 (19)
C2—C5	1.384 (2)	C8—O2	1.4568 (18)
C2—H2A	0.9300	C8—C9	1.479 (3)
C3—C4	1.3883 (18)	C8—H8A	0.9700
C3—H3A	0.9300	C8—H8B	0.9700
C4—C6	1.3890 (19)	C9—H9A	0.9600
C4—H4A	0.9300	C9—H9B	0.9600
C5—C6	1.3896 (19)	C9—H9C	0.9600
C5—H5A	0.9300	N1—N1^i^	1.250 (2)
			
C3—C1—C2	120.05 (12)	O1—C7—O2	124.33 (14)
C3—C1—N1	115.19 (12)	O1—C7—C6	123.40 (14)
C2—C1—N1	124.76 (12)	O2—C7—C6	112.27 (12)
C5—C2—C1	119.41 (13)	O2—C8—C9	110.67 (16)
C5—C2—H2A	120.3	O2—C8—H8A	109.5
C1—C2—H2A	120.3	C9—C8—H8A	109.5
C1—C3—C4	120.31 (12)	O2—C8—H8B	109.5
C1—C3—H3A	119.8	C9—C8—H8B	109.5
C4—C3—H3A	119.8	H8A—C8—H8B	108.1
C3—C4—C6	119.77 (12)	C8—C9—H9A	109.5
C3—C4—H4A	120.1	C8—C9—H9B	109.5
C6—C4—H4A	120.1	H9A—C9—H9B	109.5
C2—C5—C6	120.71 (13)	C8—C9—H9C	109.5
C2—C5—H5A	119.6	H9A—C9—H9C	109.5
C6—C5—H5A	119.6	H9B—C9—H9C	109.5
C4—C6—C5	119.73 (12)	N1^i^—N1—C1	114.02 (14)
C4—C6—C7	122.27 (12)	C7—O2—C8	117.50 (13)
C5—C6—C7	117.99 (12)		
			
C3—C1—C2—C5	1.4 (2)	C4—C6—C7—O1	177.29 (15)
N1—C1—C2—C5	−178.86 (12)	C5—C6—C7—O1	−2.0 (2)
C2—C1—C3—C4	−1.5 (2)	C4—C6—C7—O2	−2.41 (19)
N1—C1—C3—C4	178.77 (11)	C5—C6—C7—O2	178.28 (12)
C1—C3—C4—C6	0.1 (2)	C3—C1—N1—N1^i^	179.91 (13)
C1—C2—C5—C6	0.0 (2)	C2—C1—N1—N1^i^	0.2 (2)
C3—C4—C6—C5	1.2 (2)	O1—C7—O2—C8	−0.7 (2)
C3—C4—C6—C7	−178.06 (12)	C6—C7—O2—C8	179.05 (12)
C2—C5—C6—C4	−1.3 (2)	C9—C8—O2—C7	−91.4 (2)
C2—C5—C6—C7	178.04 (12)		

Symmetry codes: (i) −*x*+2, −*y*, −*z*+2.
